# Pathologic complete response after neoadjuvant therapy for locally advanced rectal cancer in a real-world setting: a population-based study

**DOI:** 10.3389/fonc.2025.1573819

**Published:** 2025-05-30

**Authors:** Lina Cadili, Jonathan M. Loree, Michael Peacock, Kimberly DeVries, Amandeep Ghuman, Ahmer A. Karimuddin, P. Terry Phang, Manoj J. Raval, Carl J. Brown

**Affiliations:** ^1^ Division of General Surgery, Department of Surgery, Faculty of Medicine, University of British Columbia, Vancouver, BC, Canada; ^2^ Division of Medical Oncology, Department of Medicine, Faculty of Medicine, University of British Columbia, Vancouver, BC, Canada; ^3^ Division of Radiation Oncology and Developmental Therapeutics, Department of Surgery, Faculty of Medicine, University of British Columbia, Vancouver, BC, Canada; ^4^ Cancer Surveillance & Outcomes, Data & Analytics, British Columbia Cancer Agency, Vancouver, BC, Canada; ^5^ Providence Health Care, University of British Columbia, Vancouver, BC, Canada

**Keywords:** colorectal cancer, rectal cancer, pathologic complete response (pCR), neoadjuvant therapy, locally advanced rectal cancer, oncology

## Abstract

**Aim:**

This study aimed to determine the impact of time from neoadjuvant therapy (NAT) to surgery on the complete pathologic response (pCR) rate in patients with locally advanced rectal cancer. NAT decreases the local recurrence of rectal cancer. Some patients achieve a pCR. The optimal time between NAT and surgery to maximize pCR remains uncertain.

**Method:**

We identified adults with T_any_, N_any_, M_0_ rectal adenocarcinoma treated with short-course radiation therapy (SCRT) or long-course chemoradiotherapy (LCRT) followed by total mesorectal excision. Multivariable logistic regression examined characteristics associated with pCR and survival.

**Results:**

In total, 3,476 patients were included from between 2000 and 2017. Of these, 1,554 (44.7%) received LCRT and 1,796 (51.7%) SCRT. The pCR rate was 13.2% (181/1373) among the LCRT group and 1.5% (26/1770) among the SCRT group. A pCR among the SCRT group was positively associated with weeks from SCRT to surgery [odds ratio (OR) 1.45, 95% confidence interval (CI) 1.13,1.86; p=0.003], tumor grade (grade 1 OR 5.72, 95% CI 1.70, 19.30, p=0.005), and stage (stage 1 OR 7.07, 95% CI 2.49, 20.08, p=<0.001). The pCR rate among the LCRT group was not associated with weeks from LCRT to surgery but was associated with sex and stage. Median follow-up was 9.5 years, and median overall survival (OS) was 9.7 years. Among patients receiving LCRT, the 5-year OS rate was higher (69.8%) when surgery followed LCRT by 6–10 weeks compared to those undergoing surgery <6 weeks or 10+ weeks post-LCRT (*p* = .003).

**Conclusion:**

Among rectal cancers treated with LCRT in a population-based cohort, longer delay to radical resection is associated with increased pCR rate. However, the overall pCR rate was lower than that reported in trial populations.

## Introduction

Rectal cancer is the third most diagnosed cancer across North America (1). One in 14 Canadian men and 1 in 18 Canadian women will develop rectal cancer during their lifetime (1). Surgery plays a critical role in the treatment of rectal cancer. However, surgery alone results in local recurrence in up to 55%–65% of low, locally advanced tumors (2). Locally recurrent disease is often incurable and causes significant morbidity (3). Neoadjuvant chemoradiation is known to reduce the local recurrence of rectal cancer (2, 4). Compared to post-operative chemoradiation, pre-operative chemoradiation is associated with improved compliance, improved local control, reduced toxicity, and better sphincter preservation in patients with low-lying tumors (5).

A pathologic complete response (pCR) is defined as the absence of cancer cells in the surgical resection specimen after neoadjuvant therapy (i.e., ypT0N0) (7). The impact of a pCR on local recurrence after surgery is controversial, but most studies suggest improved local recurrence in this cohort (8). A pCR occurs in 15%–27% of patients after chemoradiation and 1%–10% after radiotherapy alone, and some studies have shown a survival advantage in patients treated with neoadjuvant therapy (4, 6). Further, recent enthusiasm for “watch and wait” strategies, where surgery is deferred when a clinical complete response is observed, means that an improved understanding of the timing and predictors of a pCR are more relevant than ever. Chemoradiation continues to be an important treatment modality, as some patients, such as older or frail patients, may not be candidates for total neoadjuvant therapy.

In British Columbia, Canada, provincial clinical guidelines recommend neoadjuvant radiotherapy in patients with clinical stage II/III tumors (9). Neoadjuvant therapy can be administered as a short course (SCRT) with surgery recommended within 1 week or as a long course combined with chemotherapy (LCRT), where surgery is typically performed 4–8 weeks after treatment completion (10). However, in clinical practice, the time from radiotherapy completion to curative intent surgery is variable. After completion of chemoradiation, the optimal time to surgery to maximize the chances of pCR in the general population of patients with rectal cancer is uncertain. The primary objective of our study was to determine the association between time to surgery and pCR after SCRT and LCRT in a provincial population-based cohort. Our secondary objectives included a comparison of the effect of SCRT and LCRT on a pCR, predictors of a pCR, and the association between a pCR and long-term oncologic outcomes.

## Methods

### Data source

In British Columbia, Canada, a prospectively maintained colorectal cancer database through British Columbia Cancer (BC Cancer) maintains clinical, pathologic, treatment, and outcome data for all patients referred for radiation or chemotherapy treatment. In this province, BCC is the only provider of radiotherapy for patients with rectal cancer. All adult patients with histologically proven rectal adenocarcinoma treated by SCRT or LCRT followed by total mesorectal excision from 2000 to 2017 were identified from this database to ensure a minimum of 5 years of follow up at the time of analysis.

### Population

The eligibility criteria for this study were as follows: (1) histologically confirmed adenocarcinoma of the rectum in adult patients (>18 years of age); (2) T(any), N(any), M0 confirmed on CT/MRI; (3) patients who completed neoadjuvant therapy (NAT), with either SCRT or LCRT, followed by total mesorectal excision (TME). We excluded extreme/outlier patients, such as those with incomplete SCRT/LCRT, patients with unusually long delays in time to surgery, and patients who started chemotherapy more than a month before radiation therapy (thus, we excluded patients who may have had a full course of chemotherapy). This is because we were interested in studying the ‘typical’ patient and examined a more homogenous group of patients to try to understand the association between the timing of the surgery and the outcomes of interest. Data including demographic information, tumor characteristics, type and timing of neoadjuvant therapy, timing of surgery, overall survival (OS), disease-free survival (DFS), and local recurrence (LR) were collected prospectively.

### Treatment and evaluation

The SCRT group was treated with 2,500 cGy in 5 fractions over 5 days. The LCRT group was treated with 4,500 cGy in 25 fractions over 5 weeks, with a possible boost of 540 cGy in 3 fractions. The LCRT group also received one of the following chemotherapeutic regimens concurrently with radiation therapy: (1) 5-fluorouracil, (2) oxaliplatin, or (3) capecitabine. Following these treatments, all the patients had TME in the form of a low anterior resection, abdominoperineal resection, or low Hartmann’s procedure. During the study period, the time to surgery recommended by BCC was 1 week after SCRT and 4–6 weeks after LCRT. However, the timing of surgery after SCRT or LCRT was decided by the treating surgeon.

### Definition of outcomes

Staging is defined by the 7^th^ edition American Joint Committee on Cancer criteria, given the era of the included patients. The primary outcome is a pCR, defined as the absence of tumor cells in the surgical specimen (ypT0N0). Downstaging is defined as any downstage in T and N stage, any downstage in T and N- or N stage remains the same at N+, or any downstage in N (with T stage remaining the same or increasing). Secondary endpoints include overall survival, disease-free survival, and local recurrence outcomes. OS is defined as from the date of diagnosis to the date of death from any cause. DFS is defined as the interval between the date of diagnosis and the date of first local, regional, or distant recurrence, or death from colorectal cancer.

### Statistical analysis

A complete response analysis was performed on the SCRT and LCRT groups separately. Multivariable logistic regression was used to examine demographic and tumor features associated with a pCR. The covariates included in the models were specified *a priori* and include age at diagnosis, sex, tumor grade, tumor location, and number of weeks from SCRT or LCRT to surgery. pCR was a binary outcome in the model. For the complete response regression model, the number of weeks from LCRT to surgery was fit with a restricted cubic spline with three knots at 5, 6.9, and 10.3 weeks and for SCRT to surgery with three knots at 0.4, 1.1 and 2.1 weeks; these splines allow non-linear relationships to be modeled and better fit the data.

Survival outcomes were estimated using the Kaplan–Meier method. OS between patients differing by type of NAT and number of weeks from NAT to surgery (categorized) were compared using the log-rank test. Competing risk analysis was used to evaluate DFS. Patients without recurrence were censored at the date of last follow-up and death from other causes was classified as a competing event. The Gray test was used to compare the incidence of any recurrence or death from colorectal cancer between patients differing by type of NAT and number of weeks from NAT to surgery.

Multivariable analysis with Cox regression modeling was used to assess patient, clinical, and treatment factors associated with OS, and Fine–Gray modeling was used to associate DFS with patient, clinical, and treatment factors. Multivariable Cox and Fine–Gray regression models were fit, adjusting for age at diagnosis, sex, tumor grade, tumor location, type of NAT (SCRT or LCRT), and number of weeks from NAT to surgery. Cumulative incidence rates were used to analyze DFS outcomes, as we were investigating competing risks. The Kaplan–Meier method was used to analyze time to local recurrence. A p-value of ≤0.05 was considered statistically significant.

SAS 9.4 and R 4.2.1 were used for the statistical analysis.

## Results

**Figure 1:**
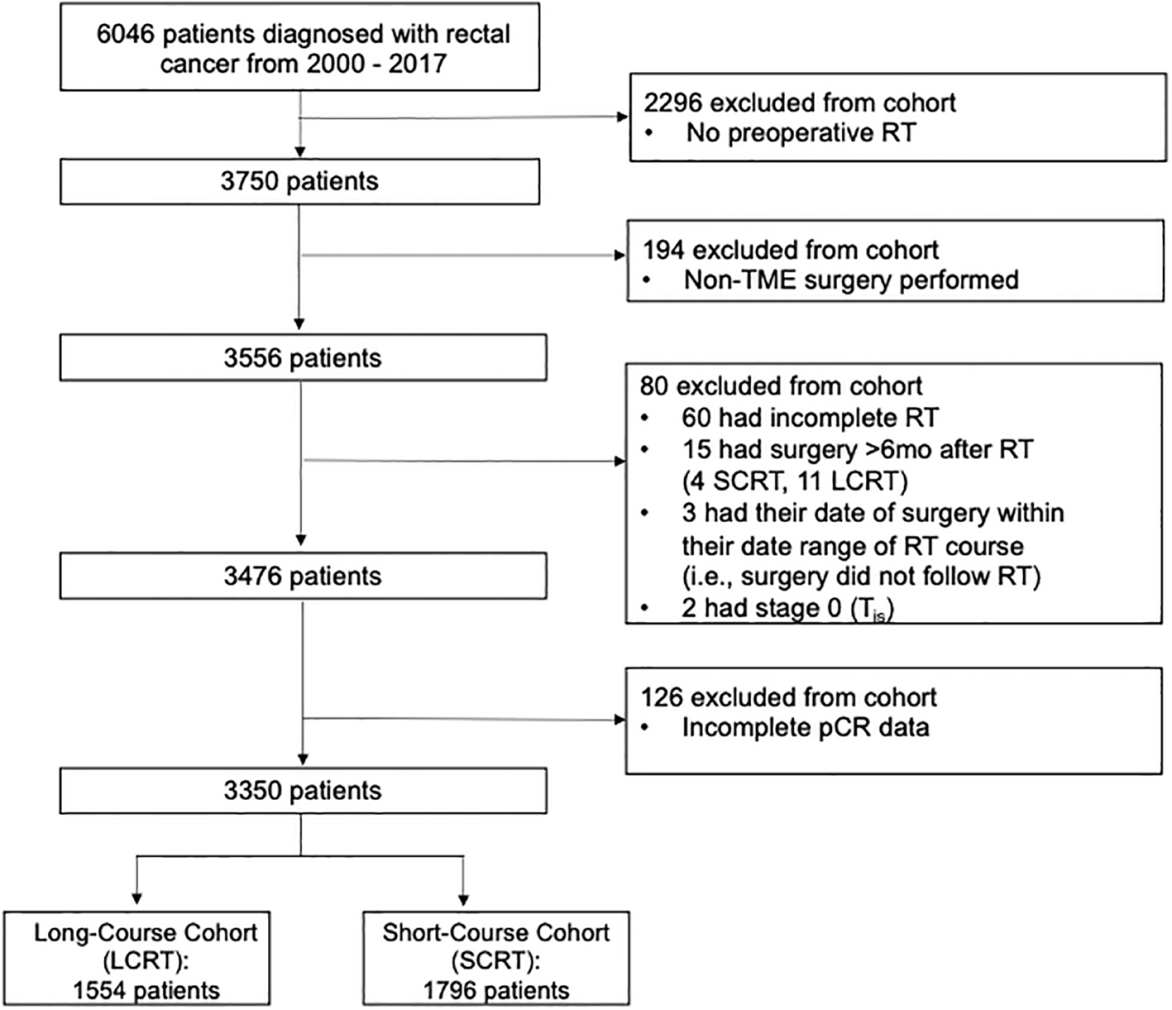
Flowchart of patients in each cohort after applying inclusion and exclusion criteria.

A total of 6,046 patients were diagnosed with rectal cancer and referred to BC Cancer between 2000 and 2017. Of the 6,046 rectal cancer patients identified, 3,350 were included in the study after the exclusion criteria were applied: 1,554 in the LCRT group and 1,796 in the SCRT group. Of these, 3,476 patients met the inclusion criteria. Survival analysis was performed on these 3,476 patients. A complete response analysis was performed on 3,350 patients because 126 patients had incomplete pCR data in the database (**Figure 1**).

The patient characteristics are displayed in **Table 1**. Most of the patients underwent an anterior resection (59.7%) or abdominoperineal resection (39.2%) (**Table 2**). The overall pCR rate was 6.2% (207/3350) (**Table 1**). Among the LCRT cohort, the pCR rate was 13.2% (181/1373) and among the SCRT cohort, the pCR rate was 1.5% (26/1770) (**Table 1**). Among the full cohort, 1,199 were downstaged (35.8%) and 1169 (34.9%) had no change in their staging following neoadjuvant therapy (**Table 2**).

**Table 1:** Patient, tumour, and treatment characteristics by pathologic complete response.

	Pathologic Complete	Response (pCR)
	No n=3143 n (%)	Yes n=207 n (%)
Age		
Median (IQR)	65 (57-74)	62 (29-88)
Sex		
Female	1073 (34.1)	85 (41.1)
Male	2070 (65.9)	122 (58.9)
Overall Stage		
1	255 (8.1)	15 (7.3)
2	936 (29.8)	77 (37.2)
3	1943 (61.8)	113 (54.6)
Unknown	9 (0.3)	2 (1)
Grade		
1	198 (6.3)	17 (18.2)
2	2260 (71.9)	104 (50.2)
3/4	354 (11.3)	8 (3.9)
Unknown	331 (10.6)	78 (37.7)
Tumor Location		
Distal	828 (26.3)	20 (9.7)
Mid	1646 (52.4)	62 (29.9)
Upper	310 (9.9)	114 (55.1)
Unknown	359 (11.4)	11 (5.3)
Neoadjuvant Therapy		
LCRT	1373 (43.7)	181 (87.4)
SCRT	1770 (56.3)	26 (12.6)

IQR, Interquartile range; LCRT, Long course chemoradiotherapy; RT, Short course radiotherapy.

**Table 2:** Type of surgery and proportion of patients with complete response or change in stage.

Surgery Type	n (%)
Anterior Resection	2076 (59.72)
Abdominoperineal Resection	1361 (39.15)
Hartmann’s Procedure	25 (0.72)
Type of Resection Not Specified	14 (0.40)
Response Type	n (%)
Complete response	207 (6.2)
Downstaged	1199 (35.8)
No change	1169 (34.9)
Upstaged	157 (4.7)
Unknown	618 (18.4)
Unknown T or N at clinical stage	584 (17.4)
Unknown T or N at pathological stage	34 (1.0)

### Pathologic complete response

**Figure 2:**
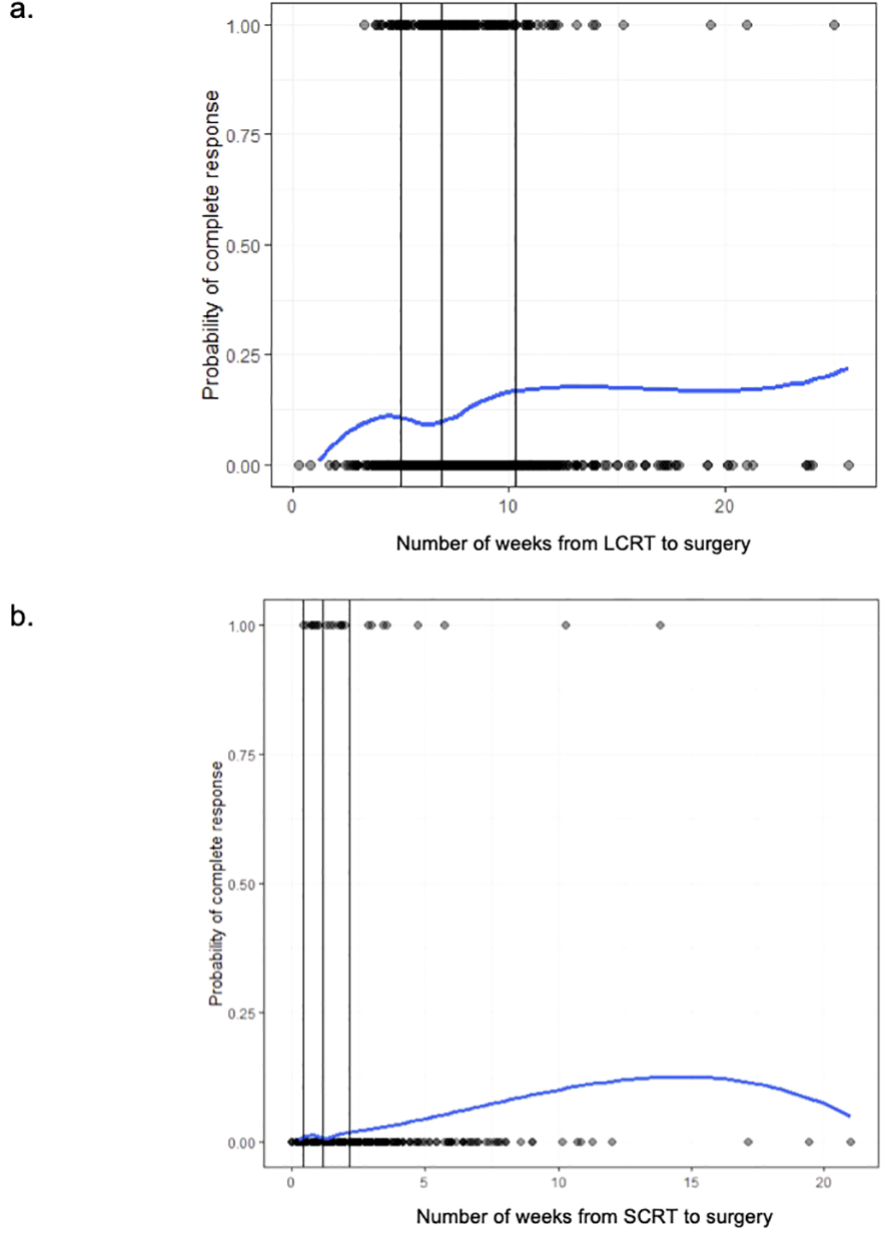
**(a)** The probability of pathologic complete response (1 = pCR, 0 = no pCR) among long-course (LCRT) patients based on the time (in weeks) from completion of LCRT to surgery. **(b)** The probability of pathologic complete response (1 = pCR, 0 = no pCR) among short-course (SCRT) patients based on the time (in weeks) from completion of SCRT to surgery.

Among the LCRT group, there appeared to be an increased chance of a pCR as the number of weeks from completion of LCRT to surgery increased, with a plateau after approximately 10 weeks (**Figure 2a**). From the smoothed line of probability, the relationship between the probability of a pCR and the number of weeks from completion of LCRT to surgery appeared to be non-linear. The vertical lines are the cubic spline knot locations (5, 6.9, and 10.3 weeks), so the effect (slope) is allowed to vary between those regions rather than being entirely linear across all the values of days from LCRT to surgery. From the adjusted logistic regression analysis, the number of weeks from completion of LCRT to surgery was not statistically associated with a complete response (**Table 3**). The odds of the probability of a pCR after adjusting for days from completion of LCRT (using a value of 10 weeks compared to a reference value of 6 weeks) to surgery were 1.25 (95% CI 0.97,1.62; p=0.084) (**Table 3**). Patient sex and stage were associated with a complete response. The odds of the probability of a pCR after adjusting for female sex was 1.41 (95% CI 1.01, 1.96, p=0.041) and after adjusting for stage 2 compared to 3 was 2.10 (95% CI 1.48, 2.97, p=<0.001).

**Table 3:** Adjusted logistic regression analysis for odds of pathologic complete response among long-course (LCRT) patients.

Covariate	Value	Reference	Odds Ratio (95% CI)	Estimate (log odds)	Standard Error	p- value
**Age at Diagnosis**	1 year	None	0.99 (0.98,1.01)	-0.01	0.01	0.193
Weeks LCRT to Surgery^a^	10	6	1.25 (0.97,1.62)	0.23	0.13	0.084
**Sex**	F	M	1.41 (1.01,1.96)	0.34	0.17	**0.041**
**Grade**	1	3/4	1.59 (0.62,4.12)	0.47	0.48	0.337
	2	3/4	0.95 (0.44,2.05)	-0.05	0.39	0.899
	Unknown	3/4	3.25 (1.48,7.16)	1.18	0.40	**0.003**
**Stage**	1	3	1.29 (0.53,3.16)	0.25	0.46	0.577
	2	3	2.10 (1.48,2.97)	0.74	0.18	**<0.001**
**Tumor Location**	Distal	Mid	0.76 (0.53,1.10)	-0.27	0.19	0.143
	Upper	Mid	0.62 (0.31,1.26)	-0.47	0.36	0.188
	Unknown	Mid	0.49 (0.29,0.84)	-0.71	0.27	**0.009**

a Has a non-linear component, so this does not fully convey odds of complete response. Bolded numbers are statistically significant.

Among the SCRT patients, there appeared to be higher odds of a pCR as the number of weeks from completion of SCRT to surgery increased, with a plateau after approximately 12.5 weeks (**Figure 2b**). From the adjusted logistic regression analysis, the number of weeks from completion of SCRT to surgery was associated with a complete response (**Table 4**). The odds of the probability of a pCR after adjusting for days from completion of SCRT (using a value of 4 weeks compared to a reference value of 2 weeks) to surgery were 1.45 (95% CI 1.13,1.86; p=0.003) (**Table 3**). Grade and stage were also associated with a complete response. The odds of the probability of a pCR after adjusting for grade 1 compared to 2 was 5.72 (95% CI 1.70, 19.30, p=0.005) and after adjusting for stage 1 compared to 3 was 7.07 (95% CI 2.49, 20.08, p=<0.001).

**Table 4:** Adjusted logistic regression analysis for odds of pathologic complete response among short-course (RT) patients.

Covariate	Value	Reference	Odds Ratio (95% CI)	Estimate (log odds)	Standard Error	p- value
**Age at Diagnosis**	1 year	None	0.97 (0.94,1.01)	-0.03	0.02	0.126
Weeks SCRT to Surgery^a^	4	2	1.45 (1.13,1.86)	0.37	0.13	**0.003**
**Sex**	F	M	1.63 (0.74,3.60)	0.49	0.40	0.224
Grade^b^	1	2	5.72 (1.70,19.30)	1.74	0.62	**0.005**
	Unknown	2	16.12 (6.56,39.63)	2.78	0.46	**<0.001**
**Stage**	1	3	7.07 (2.49,20.08)	1.96	0.53	**<0.001**
	2	3	2.59 (0.94,7.16)	0.95	0.52	0.067
**Tumor Location**	Distal	Mid	0.77 (0.28,2.08)	-0.27	0.51	0.603
	Upper	Mid	0.27 (0.04,2.06)	-1.30	1.03	0.208
	Unknown	Mid	0.44 (0.06,3.29)	-0.83	1.03	0.421

a Has a non-linear component, so this is not fully convey odds of complete response.

b 258 cases with grade ¾ were excluded from the analysis of grade as there are no cases with the outcome event pCR. Bolded numbers are statistically significant.

### Overall survival

**Figure 3:**
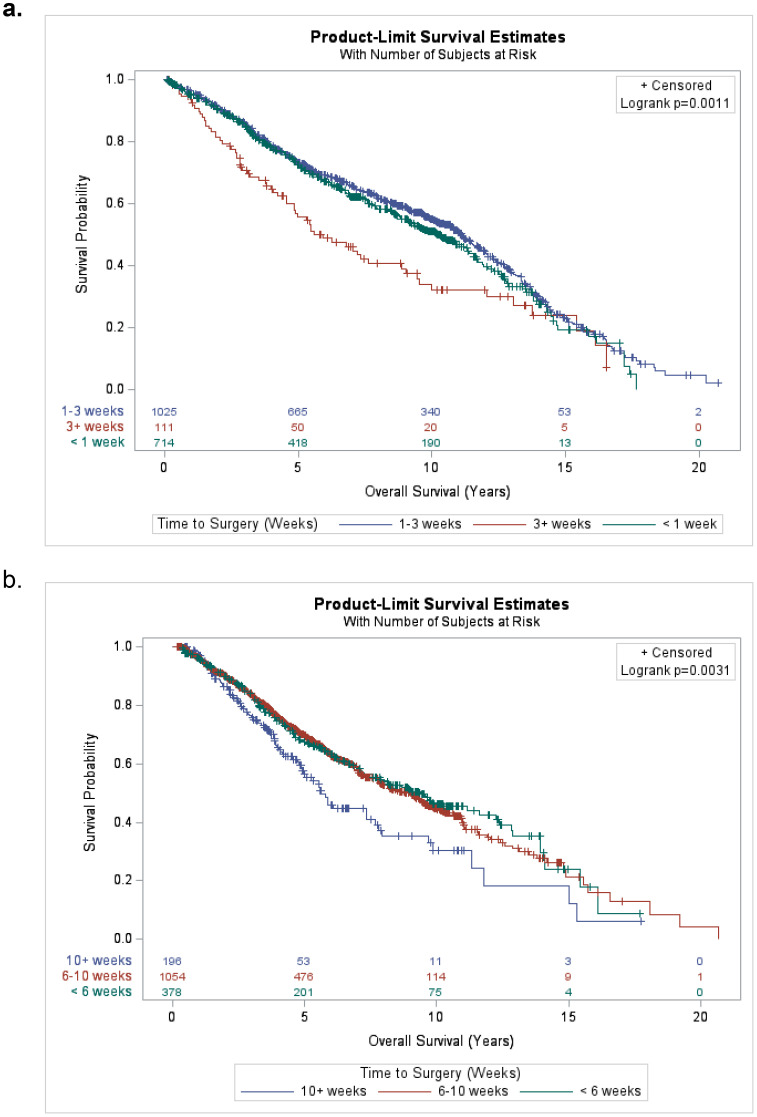
**(a)** Kaplan-Meier plot for overall survival of short-course (SCRT) patients stratified by time from completion of SCRT to surgery groups (<1 week, 1-3 weeks, and 3+ weeks). **(b)** Kaplan-Meier plot for overall survival of long-course (LCRT) patients stratified by time from completion of LCRT to surgery groups (<6 weeks, 6-10 weeks, and 10+ weeks).

The median follow-up time was 9.5 years (95% CI 9.2-9.7 years) and the range in follow-up was 0.07–20.7 years (IQR: 2.9-9.6 years). The median overall survival time was 9.7 years (95% CI 9.2–10.3 years). The Kaplan–Meier OS estimates at 3, 5, and 10 years are 84% (95% CI 82.5%–85%), 70.1% (95% CI 68.4%–71.7%), and 49% (95% CI 47%–51%), respectively. Stratified by time from SCRT to surgery groups, the 5-year OS rates for the SCRT patients were 72.6% (95% CI 69%–75.9%), 73.2% (95% CI 70.3%–75.9%), and 55.7% (95% CI 45.3%–64.8%) for <1 week, 1–3 weeks and 3+ weeks, respectively (p=0.001) (**Figure 3a**). Stratified by time from LCRT to surgery groups, the 5-year OS rates for the LCRT patients were 67.3% (95% CI 62%–72.1), 69.8% (95% CI 66.5%–72.7%), and 56.5% (95% CI 47.7%–64.3%) for <6 weeks, 6–10 weeks, and 10+ weeks, respectively (p=0.003) (**Figure 3b**).

From the adjusted Cox regression analysis, the number of weeks from completion of NAT to surgery, age at diagnosis, sex, tumor grade, and stage were associated with OS (**Supplementary Table S5**). The hazard ratio for OS after adjusting for age at diagnosis was 1.05 (95% CI 1.04,1.05; p<0.001), female sex was 0.85 (95% CI 0.76,0.94; p=0.003), tumor grade 2 was 0.72 (95% CI 0.62,0.83; p=<0.001), stage 1 was 0.55 (95% CI 0.45,0.66; p=<0.0.001), and stage 2 was 0.73 (95% CI 0.66,0.82; p=<0.0.001) (**Supplementary Table S5**).

### Disease-free survival

The 5-year cumulative incidence rates of disease progression/death were 8.6% (95% CI 5.1–13.2) and 30.3% (95% CI 28.6–31.9) for a pCR vs. no pCR, respectively. There was a statistically significant difference in the cumulative incidence rates for DFS by pCR, meaning there were higher cumulative incidence rates of recurrent disease/rectal cancer death among the patients who did not have pCR vs. those who did (p<0.0001) (**Supplementary Figure S4**). The association remained the same if we focused on the patients who received LCRT. Stratifying by stage, the 5-year cumulative incidence rates of disease progression/death were 14.3% (95% CI 10.4–18.9), 19% (95% CI 16.6–21.5), and 36.2% (95% CI 34–38.3) for stages 1, 2, and 3, respectively. There was a statistically significant difference in the cumulative incidence rates for DFS by overall stage of disease (with higher cumulative incidence rates of recurrent disease/colorectal cancer death associated with increasing stage of disease) (p<0.0001) (**Supplementary Figure S5**). Further analyses were performed after stratifying for both staging and pCR. There was a statistically significant difference in the cumulative incidence rates for DFS by overall stage of disease for patients who did not have a pCR (p<0.0001) and there was no statistically significant difference in DFS by stage for patients with a pCR (p=0.337) (**Supplementary Figures S8, S9**).

From the adjusted Fine–Gray competing risk regression analysis, age, number of weeks from completion of NAT to surgery, grade, stage, and tumor location were associated with disease-free survival (**Supplementary Table S6**). When including the non-linear component of weeks of SCRT to surgery, the association with DFS was not significant (p = 0.967), but if we analyzed it as linear, the association with DFS became significant when using the Wald Chi-Square test (p=0.014) (**Supplementary Table S7**).

### Local recurrence

There were 41 local recurrences among the 3476 patients analyzed. There was no statistically significant difference in local recurrence stratified by pCR (p=0.120) (**Supplementary Figure S6**) or by stage (p=0.269) (**Supplementary Figure S7**). There were no local recurrences in the patients who had a pCR. In the patients who did not have a pCR, there was no statistically significant difference in LR by stage (p=0.371) (**Supplementary Figure S10**).

## Discussion

The combined treatment modalities of neoadjuvant therapy with TME have greatly reduced local recurrence rates after R0 resection for locally advanced rectal cancer and have improved outcomes (14). Several studies have shown that the outcomes of patients with a pathologic complete response after neoadjuvant therapy are better than those without a pathologic complete response (14). Wasmuth et al. reviewed 1,384 patients and found the estimated 5-year OS to be 87% among those with a pCR, and 67% among those without a pCR (15). In our review of 3,350 patients, 6.2% of patients had a pCR and 35.8% of patients were downstaged after neoadjuvant therapy. Among the long-course cohort, the pCR rate was 13.2% (181/1373) and among the short-course cohort, the pCR rate was 1.5% (26/1770).

The pCR rate following LCRT in the literature ranges from 15% to 27%; our rate of 13.2% was slightly lower, likely because most of our patients had surgery within 4 weeks of treatment, particularly in the early years of the 2000–2017 study period. Furthermore, patients in British Columbia were typically recommended LCRT in this timeframe if they had more locally advanced tumors. We expect that our provincial pCR rates have increased as our understanding of delayed surgery has evolved. Most patients had recommended surgery within a few days after completion of their SCRT, so we would expect their pCR to be low. As most of these patients had T3N0 disease, we expect that they would have had a much higher pCR rate had they been treated with long-course therapy. Our results are in keeping with the literature, as most studies have found a lower pCR rate among patients treated with SCRT compared to LCRT (16).

We do not believe that changes in treatment protocols over the 17-year span of our study greatly affected the results. All patients during this era would have been treated with a computer tomography (CT)-based radiation planning. There was an increase in use of volumetric modulated arc therapy (VMAT) compared to threedimensional radiation therapy (3DRT), but the doses within the target area (45 Gy-50.4 Gy) would have remained fairly consistent, and the difference in technique would have been expected to lower the dose to organs at risk (OARs) outside the target area rather than increase tumour response. Adjuvant therapy would not have affected the pCR rates and neoadjuvant chemotherapy would not have been routinely offered during this time.

The ideal timing of surgery following neoadjuvant therapy is an ongoing debate. Increasing the interval to surgery leads to an increased pCR rate by facilitating prolonged tumor necrosis (7). Several studies have shown that delaying surgery 8 weeks is safe and leads to higher rates of pCR (17, 18). Tulchinsky et al. found that increasing the interval to surgery more than 7 weeks improved the pCR rate to 35% (compared to 17% for patients having surgery within 7 weeks), and Sloothaak et al. found that delaying surgery 15–16 weeks after radiotherapy resulted in the highest pCR rate of 18% compared to other time intervals they studied (11, 12). A National Institutes of Health study showed that increasing the time interval between neoadjuvant therapy to surgery to an average of 11 weeks increased the pCR rate to 25% without excess surgical complication (19). In contrast, Huntington et al. found that a delay in surgery beyond 60 days after radiotherapy was associated with higher margin positivity and worse survival (13).

Among our population, delaying surgery after LCRT did not significantly increase the pCR rate. However, the cubic splines analysis did suggest that the pCR rate reaches stability at 10.3 weeks after NAT is complete. Thus, waiting more than 10 weeks after LCRT for surgery is potentially not beneficial for patients. Delaying surgery after SCRT did significantly increase the pCR rate. A patient treated with SCRT who had surgery 4 weeks after completion of SCRT has 1.45 times the odds of achieving a pCR compared to a patient who had surgery 2 weeks after completion of SCRT.

When the SCRT and LCRT were combined, there was a significant increase in the pCR rate with delayed surgery. It is possible that analyzing the cohorts separately affected the power of our study. Some of our patients underwent surgery more than 20 weeks after completion of SCRT, which was due to practical nuances such as delays in referral to subspecialist, medical issues such as a major myocardial infarction that would postpone surgery, patient preference, etc. It has been postulated that neoadjuvant SCRT increases sphincter-preserving surgery; however, several studies have shown that there is no correlation between pCR/delay to surgery and sphincter preservation (19–21). While not demonstrable in aggregate, there were certainly clinical scenarios where tumor downstaging facilitated surgical reconstruction in this cohort.

The 5-year OS rate for the SCRT group was highest for the < 1-week (72.6%) and 1–3-week (73.2%) groups, and lower for the 3+ weeks group (55.7%). This could be because the patients who had surgery 3+ weeks after neoadjuvant therapy had worse disease and/or were delayed due to other medical issues that perhaps ultimately impacted their OS. For the LCRT group, the 5-year OS rates were highest for the < 6-week (67.3%) and 6–10-week (69.8%) groups, and lower for the 10+ week group (56.5%). This may be due to those with worse local disease waiting longer to proceed to surgery. We found significantly higher cumulative incidence rates of recurrent disease/rectal cancer death among the patients who did not have a pCR vs. those who did. Abdul-Jalil et al. also found a pCR to be a significant predictor of DFS, and Yeo et al.’s multicenter study highlights favorable DFS outcomes in patients who achieved a pCR (22, 23).

When comparing treatment strategies and outcomes in different geographic regions, we found a variation between Western and Asian countries. A study from the Netherlands reviewed 1,009 patients from across Europe and South America; 91% of the patients underwent chemoradiation (similar protocol to the patients in our study), and 890 had a clinical complete response with an overall survival of 87.9% (24). In contrast, in Japan, radiation therapy is not used very often. As outlined by Malakron and Chang, less than 2% of rectal cancer patients with T4 disease in Japan were treated with neoadjuvant CRT in a cohort of 1,191 patients (25). This is a notable difference in treatment patterns compared to our center, highlighting the diverse treatment strategies for locally advanced rectal cancer.

Local recurrence was not found to be affected by a pCR or stage in this study; however, there were only 41 local recurrences among the 3476 patients, thus the study was likely not powered to detect an effect. Given there were no events in one of the strata (i.e., no local recurrences in the pCR strata) and the median survival time was not estimable in the time to local recurrence analysis, calculating sample size was more challenging. With a power of 80% and alpha of 0.05 and proportion of 6% of subjects with a pCR in the cohort (n=207/3476), attempting to detect a relative hazard ratio of 0.5 (pCR/no pCR) would require 290 total events. Even then, assuming a 50% benefit of a pCR for the risk of local recurrence may be optimistic. If we hypothetically assume a median survival time of 7.5 years in the non-pCR group, a censoring rate of 98% (assumed equal for both groups), and a planned average length of follow-up of 10 years, a total sample size of 3,464 subjects would be needed and we would have needed to see 9 local recurrence events occurring in the pCR group and 281 local recurrence events in the non-pCR group). Based on these estimates, it is reasonable to believe we would most likely be underpowered to detect an association between pCR and local recurrence, given one truly exists.

This study is the largest cohort study on locally advanced rectal cancer in British Columbia, Canada. The results will help inform patients and practitioners of the likelihood of achieving a pCR and potentially being offered non-operative management (NOM), especially in patients who may not be candidates for neoadjuvant chemotherapy. For patients not requiring downstaging but interested in NOM, particularly those who are not candidates for neoadjuvant chemotherapy, we would select LCRT over SCRT.

This study is limited by its retrospective nature. The database has missing information, such as incomplete pathology data for full predictive analysis. The selection of LCRT vs. SCRT and the timing of surgery after completion of LCRT/SCRT were not standardized and contingent on the surgeon’s and radiation oncologist’s practice patterns. In addition, the study period was from 2000 to 2017, and rectal cancer therapy has evolved since this time period, including the widespread adoption of total neoadjuvant therapy (TNT) in combination with both LCRT and SCRT for advanced tumors. Future research directions include reviewing patients for the last 5 years, and assessing patient outcomes with TNT.

## Conclusion

Locally advanced rectal cancer benefits from neoadjuvant therapy followed by total mesorectal excision. Higher rates of pCR were observed with a longer delay to radical resection in a population-based cohort, and a pCR was a significant predictor of disease-free survival. However, overall pCR rates were lower than those reported in trial populations, potentially lending further support to total neoadjuvant approaches in cases in which organ preservation is a priority.

## Data Availability

The raw data supporting the conclusions of this article will be made available by the authors, without undue reservation.
