# Supplementary material for: Pathologic complete response after neoadjuvant therapy for locally advanced rectal cancer in a real-world setting: a population-based study
Source: Front Oncol. 2025 May 30;15:1573819. doi: 10.3389/fonc.2025.1573819 (PMC12162324; doi:10.3389/fonc.2025.1573819)
Supplement: Supplementary file 1 [file DataSheet1.docx]

**SUPPLEMENT 1:**

**Table 5**: Adjusted Cox regression estimates for overall survival.

| **Covariate** | **Value** | **Reference** | **Hazard Ratio (95% CI)** | **Estimate (log hazard)** | **Standard Error** | **p- value** |
| --- | --- | --- | --- | --- | --- | --- |
| **Age at Diagnosis** | 1 year | None | 1.05 (1.04,1.05) | 0.04 | 0.01 | **<0.001** |
| **Weeks NAT to Surgery^a^** | 10 | 6 | 1.28 (1.17,1.41) | 0.25 | 0.05 | **<0.001** |
| **Sex** | F | M | 0.85 (0.76,0.94) | -0.16 | 0.05 | **0.003** |
| **Grade** | 1 | 3/4 | 0.75 (0.59,0.94) | -0.29 | 0.12 | 0.014 |
|  | 2 | 3/4 | 0.72 (0.62,0.83) | -0.33 | 0.07 | **<0.001** |
|  | Unknown | 3/4 | 0.74 (0.60,0.92) | -0.30 | 0.11 | **0.005** |
| **Stage** | 1 | 3 | 0.55 (0.45,0.66) | -0.60 | 0.10 | **<0.001** |
|  | 2 | 3 | 0.73 (0.66,0.82) | -0.31 | 0.06 | **<0.001** |
| **Tumor Location** | Distal | Mid | 1.00 (0.89,1.12) | 0.00 | 0.06 | 0.987 |
|  | Unknown | Mid | 1.24 (1.05,1.46) | 0.21 | 0.08 | **0.010** |
|  | Upper | Mid | 0.83 (0.69,1.01) | -0.18 | 0.10 | 0.062 |
| **NAT Group** | Long-course (LCRT) | Short-course (SCRT) | 1.04 (0.81,1.32) | 0.03 | 0.12 | 0.779 |

^a^Has a non-linear component, so this does not fully convey hazard of death.


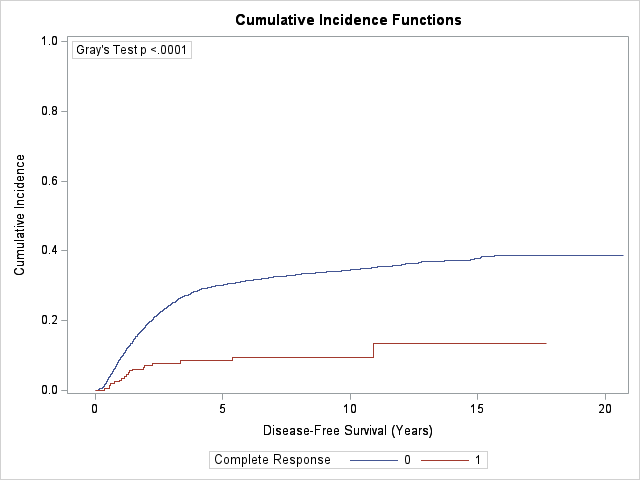


**Figure 4:** Cumulative incidence function plot for disease-free survival by time to surgery, stratified by pathologic complete response.


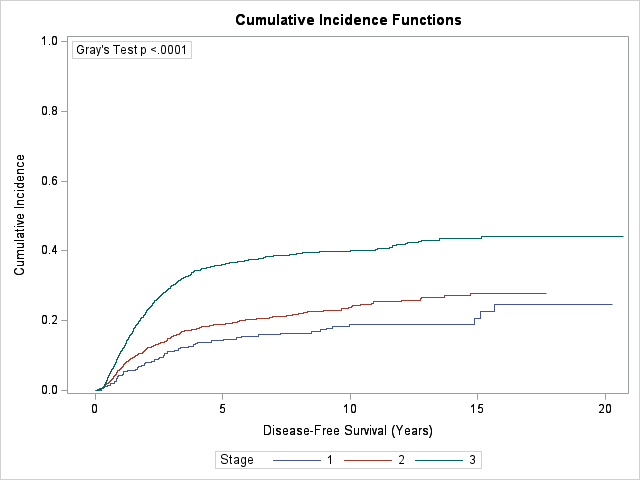


**Figure 5:** Cumulative incidence function plot for disease-free survival by time to surgery, stratified by overall stage.

**Table 6**: Adjusted Fine-Gray regression estimates for disease free survival.

| **Covariate** | **Value** | **Reference** | **Hazard Ratio (95% CI)** | **Estimate**  **(log hazard)** | **Standard Error** | **p-value** |
| --- | --- | --- | --- | --- | --- | --- |
| **Age at Diagnosis** | 1 year | None | 1.01 (1.00,1.01) | 0.01 | 0.01 | **0.040** |
| **Weeks NAT to Surgery^a^** | 10 | 6 | 1.18 (1.05,1.32) | 0.16 | 0.06 | **0.005** |
| **Sex** | F | M | 1.08 (0.95,1.23) | 0.08 | 0.06 | 0.228 |
| **Grade** | 1 | 3/4 | 0.54 (0.40,0.74) | -0.61 | 0.15 | **<0.001** |
|  | 2 | 3/4 | 0.58 (0.48,0.68) | -0.55 | 0.09 | **<0.001** |
|  | Unknown | 3/4 | 0.60 (0.47,0.76) | -0.51 | 0.12 | **<0.001** |
| **Stage** | 1 | 3 | 0.43 (0.33,0.58) | -0.83 | 0.15 | **<0.001** |
|  | 2 | 3 | 0.54 (0.47,0.63) | -0.61 | 0.08 | **<0.001** |
| **Tumor Location** | Distal | Mid | 1.10 (0.95,1.27) | 0.09 | 0.07 | 0.215 |
|  | Unknown | Mid | 1.25 (1.04,1.52) | 0.23 | 0.10 | **0.020** |
|  | Upper | Mid | 0.76 (0.60,0.98) | -0.27 | 0.12 | **0.029** |
| **NAT Group** | Long-course (LCRT) | Short-course (SCRT) | 1.04 (0.77,1.41) | 0.04 | 0.15 | 0.826 |

^a^Has a non-linear component, so this does not fully convey hazard of death.


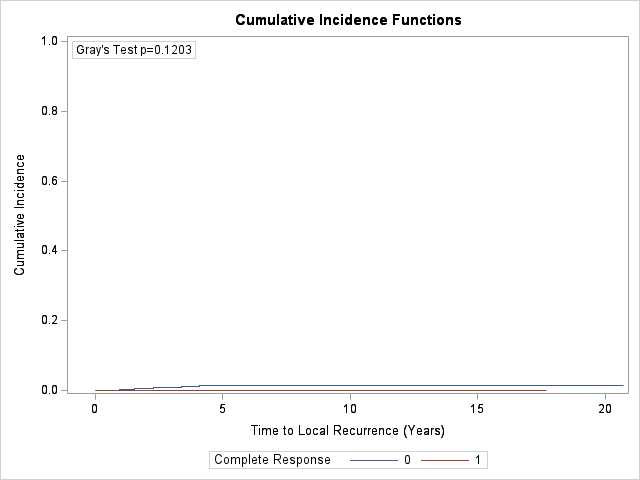


**Figure 6:** Cumulative incidence function plot for local recurrence by time to surgery, stratified by pathologic complete response.


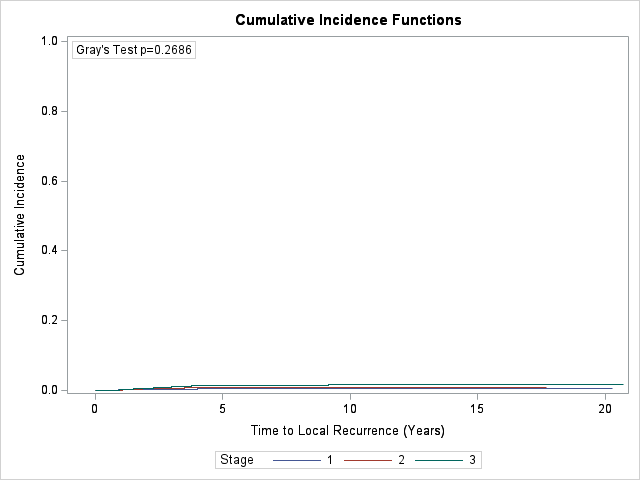


**Figure 7:** Cumulative incidence function plot for local recurrence by time to surgery, stratified by overall stage.


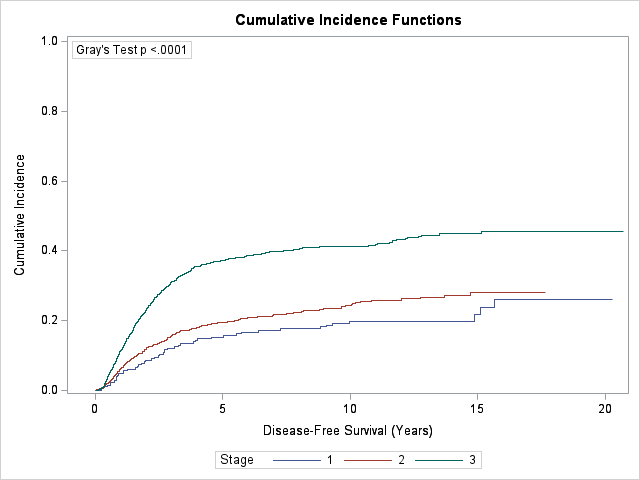


**Figure 8:** Cumulative incidence function plot for disease free survival by time to surgery, stratified by overall stage for pCR=no. The 5-year cumulative incidence rates are 15.3% (95% CI 11-20.3), 19.4% (95% CI 16.9-22.2), and 37.4% (95% CI 35.1-39.7) for stage 1, 2 and 3 respectively.


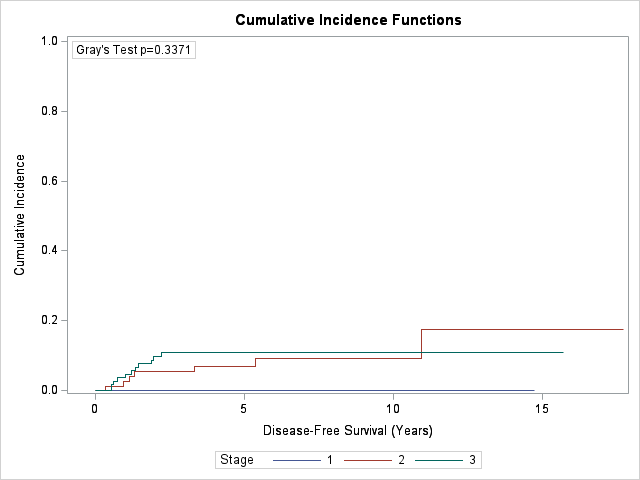


**Figure 9:** Cumulative incidence function plot for disease free survival by time to surgery, stratified by overall stage for pCR=yes. The 5-year cumulative incidence rates are 0, 7% (95% CI 2.6-14.6), and 10.9% (95% CI 5.8-18) for stage 1, 2 and 3 respectively.

**Table 7**: Wald Chi-Square test Fine-Gray competing risk regression model terms for DFS.

| **Effect** | **Wald Chi-Square** | **Degrees of Freedom** | **p-value** |
| --- | --- | --- | --- |
| **Age at Diagnosis** | 4.22 | 1 | 0.040 |
| **Sex** | 1.45 | 1 | 0.228 |
| **Weeks NAT to Surgery** | 8.58 | 2 | **0.014** |
| Nonlinear | 0.01 | 1 | 0.967 |
| **Grade** | 39.54 | 3 | **<0.001** |
| **Stage** | 84.30 | 2 | **<0.001** |
| **Tumor Location** | 12.78 | 3 | **0.005** |
| **SCRT Group** | 0.05 | 1 | 0.8261 |
| **Total** | 184.07 | 13 | **<0.001** |


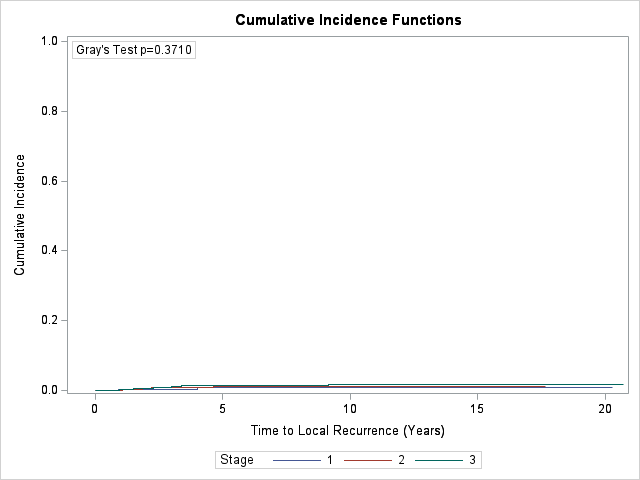


**Figure 10:** Cumulative incidence function plot for local recurrence by time to surgery, stratified by overall stage for pCR=no. The 5-year cumulative incidence rates are 0.9% (95% CI 0.2-2.9), 1.1% (95% CI 0.5-2), and 1.6% (95% CI 1.1-2.3) for stage 1, 2 and 3 respectively.
